# Catheter Ablation of Atrial Tachycardia after Pulmonary Vein Isolation in a Patient with Common Ostium of Inferior Pulmonary Veins: Case Report

**DOI:** 10.3390/medicina60020264

**Published:** 2024-02-02

**Authors:** Milos Babic, Branko Djurdjevic, Dejan Vukajlovic, Mihailo Jovicic, Masa Petrovic, Jelena Kljajevic, Milosav Tomovic, Aleksandra Nikolic

**Affiliations:** 1 Institute for Cardiovascular Diseases “Dedinje”, 11040 Belgrade, Serbia; 2 Faculty of Medicine, University of Belgrade, 11000 Belgrade, Serbia

**Keywords:** atrial fibrillation, atrial tachycardia, arrythmia, catheter ablation

## Abstract

*Background and Objectives*: Atrial fibrillation (AF), a prevalent cardiac arrhythmia, significantly impacts the quality of life of those affected. The preferred treatment for symptomatic AF, particularly when pharmacological methods fall short, is catheter ablation with pulmonary vein isolation (PVI). While common pulmonary vein (PV) anatomical variants, such as the right accessory pulmonary vein and the common ostium of left pulmonary veins (LCPV), have been studied extensively, their impact on the long-term outcome of PVI is known to be minimal. However, data on less common anomalies, like the common ostium of the left and right inferior pulmonary vein (CIPV), remain scarce in the medical literature. This report aims to shed light on the challenges and outcomes of catheter ablation in a patient with a rare CIPV anomaly. By presenting this case, we contribute to the limited knowledge about the management of such unique anatomical variations in AF treatment and discuss the importance of individualized treatment approaches. *Case Presentation*: We present a case involving a 56-year-old male diagnosed with AF in 2018. Initial PVI treatment was successful, but the patient experienced symptom recurrence after three years. A preprocedural CT scan before the second ablation revealed a CIPV anomaly. During the repeat procedure, a right superior pulmonary vein (RSPV) reisolation was performed due to identified gaps in the previous ablation line. Post-procedure, the patient maintained a sinus rhythm and reported no further symptoms. *Conclusions*: This case highlights the importance of recognizing rare PV anatomies like CIPV in the effective management of AF. Tailored ablation strategies, accounting for unique anatomical conditions, can lead to successful long-term outcomes, reinforcing the need for personalized approaches in AF treatment, especially in cases involving complex anatomical variations.

## 1. Introduction

Atrial fibrillation (AF) is a prevalent cardiac arrhythmia that can significantly impact the overall quality of life of affected individuals. Catheter ablation with pulmonary vein isolation (PVI) has become the treatment of choice in symptomatic AF patients, especially when pharmacological management is insufficient [1,2]. The literature has shown that common variants of pulmonary vein (PV) anatomy, such as the right accessory pulmonary vein or common ostium of left pulmonary veins (LCPV), have no significant impact on the long-term outcome of PVI or, in the latter case, especially LCPV might even be beneficial [3,4]. However, data for other, less frequent anomalies are missing in the literature. The common ostium of the left and right inferior pulmonary vein (CIPV) is an extremely rare variant that was only reported in a few cases undergoing catheter ablation. Previous studies have suggested a less than 1% prevalence [5,6]. Thus, electrophysiologists should be aware of such PV variants and the safety and efficacy of ablation in such cases [7]. 

## 2. Case Presentation

**Figure 1:**
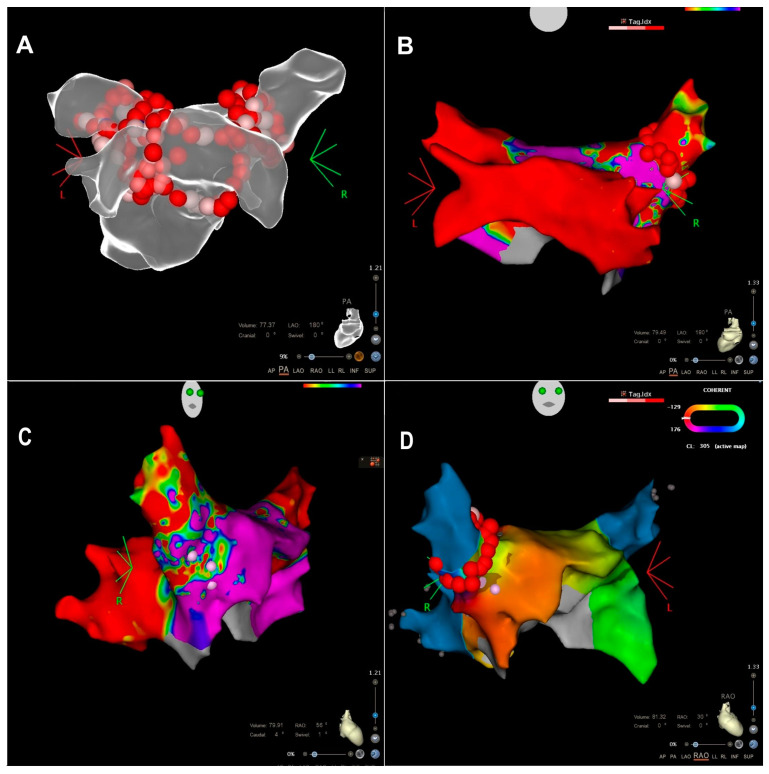
(**A**) Anatomical map of the left atrium with ablation lines during the first procedure, PA view. (**B**) voltage map from the second procedure, PA view. CIP is isolated. (**C**) voltage map, RAO view. Possible gap in the anterior/superior antral region of the RSPV. (**D**) Activation map, RAO view, with the ablation points targeting the areas responsible for the arrhythmia.

We present the case of a 56-year-old male who initially presented to our department in 2018 with symptoms of atrial fibrillation. The patient recalls having the symptoms for approximately three years. The patient is a smoker, has a BMI of 33, and has diabetes mellitus type II, which is managed with oral therapy (Glucophage 1000 mg twice daily). On echocardiographic examination, the patient had an ejection fraction of 50% without any segmental wall motion abnormalities. Following diagnosis, a PVI procedure was performed. During the procedure, “en-bloc” isolation of the lower veins was achieved, along with circumferential ablation of upper pulmonary veins and the creation of an additional line over the carina between the lower veins on the posterior wall (Figure 1A). After three years without symptoms, the patient returned to the department complaining of paroxysmal symptoms, including a daily sensation of rapid heartbeats, along with episodes of dizziness over the past few months. The patient consequently underwent cardioversion and was prescribed antiarrhythmic treatment with oral saturation of amiodaron and continued with amiodaron (200 mg once daily), bisoprolol (5 mg once daily), and rivaroxaban (20 mg once daily). Three months later, at follow-up, the patient still reported having symptoms. ECG revealed incessant atrial tachycardia (AT), with CL 300 msec and variable AV conduction, and the patient was unable to maintain sinus rhythm. As a result, catheter ablation was indicated.

**Figure 2:**
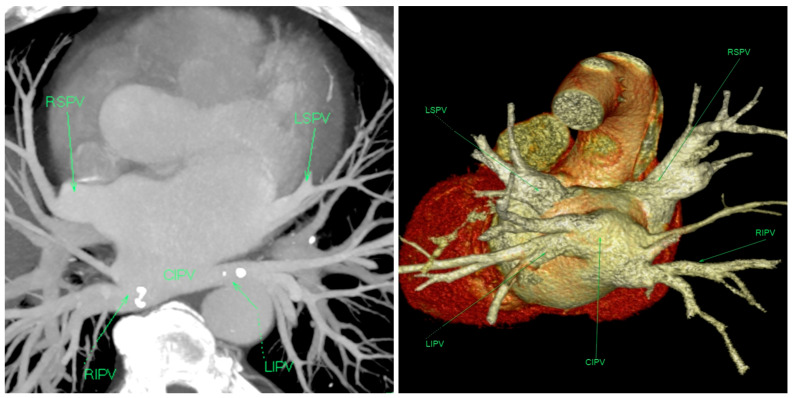
(**Left**) Standard computed tomography image projections in the modified axial view. The right and left inferior pulmonary veins can be seen entering the left atrium via an unusually large common ostium (asterisk—level of the ostium). (**Right**) The 3D volume rendered image also illustrates the common ostium of the inferior pulmonary veins.

In preparation for the repeat catheter ablation, a thorough evaluation was performed, including a repeated preprocedural CT scan of the chest, which confirmed the presence of CIPV and the absence of post-ablation PV stenosis (Figure 2). On echocardiographic examination, the only noted abnormality was the enlargement of the left atrium (48 mm in the longitudinal parasternal view). No other structural abnormalities were identified. A transesophageal echocardiography was performed that ruled out the presence of a thrombus in the left atrial appendage. 

**Figure 3:**
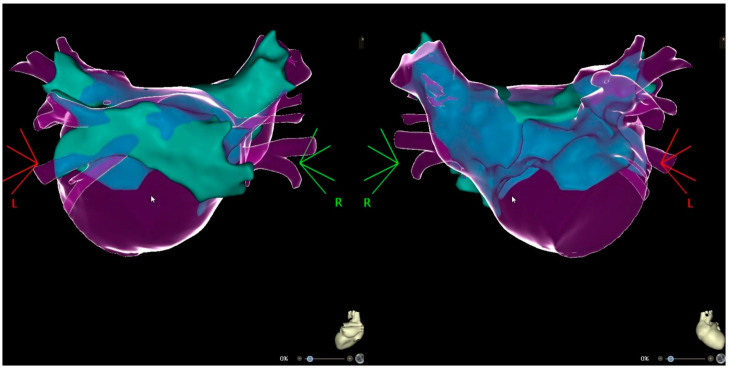
Integrated images CT scan with 3D electroanatomic map ((**Left**)—PA view; (**Right**)—AP view).

At the start of the procedure, an AT with a cycle length of 300 ms was present. Upon placement of the decapolar catheter into the coronary sinus, a double transseptal puncture was performed, guided by intracardiac echocardiography. The LA was mapped using a PentaRay multielectrode mapping catheter (CARTO3 Biosense Webster Inc., DiamondBar, CA, USA), and the ablation was performed with an irrigated contact force ablation catheter (THERMOCOOL SMARTTOUCH, Biosense Webster Inc., DiamondBar, CA, USA). The voltage map and signals documented the successful isolation of all veins except the Right Superior Pulmonary Vein (RSPV). An activation map generated by employing the Coherent module highlights the earliest activation zone in the anterior and superior antral region of the RSPV, representing the supposed gap in the place of the previous ablation. An integration image of the CT scan with the 3D electroanatomical map is depicted in Figure 3. 

**Figure 4:**
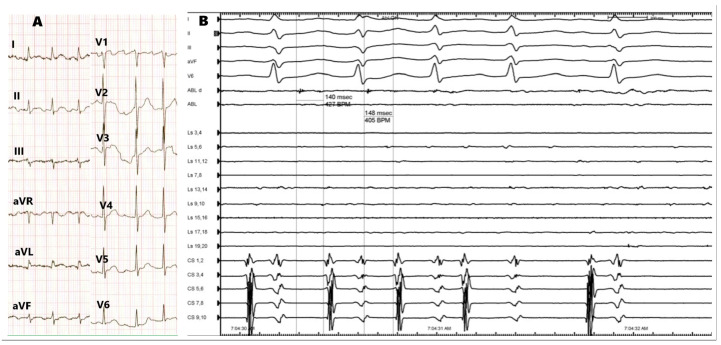
(**A**) Atrial tachycardia on the standard 12-lead ECG. (**B**) Electrograms during termination of atrial tachycardia.

A reisolation of RSPV has been performed, first leading to dissociation of the atrium, which was in sinus rhythm, from RSPV, where tachycardia continued with the same cycle length (Figure 4). Finally, after completion of the ablation line, tachycardia ceased and was not inducible during programmed atrial stimulation and burst stimulation after ablation (Figure 1B–D). The acute success was confirmed with the exit and entry block between the left atrium and PVs. The procedure was performed under moderate sedation with continuous infusion of midazolam and fentanyl. Heparin was administered based on 100 IU/kg body weight, and the activated clotting time was maintained at 300–350 s with additional doses of heparin. Considering that the origin of AT was not in the CIPV, there was no additional risk for esophagus injury, so no temperature probe was used. Our institutional protocol does not include performing gastroscopy routinely in patients after ablation for atrial fibrillation. No procedural or periprocedural complications occurred. The patient was discharged the following day in sinus rhythm and was prescribed bisoprolol (2.5 mg once daily), rivaroxaban (20 mg once daily), and esomeprazole (20 mg once daily). At three months follow-up, the patient remains well, without any complaints suggestive of tachycardia. Holter ECG analysis showed sinus rhythm without recurrence of AF/AT. After six months, a telephone follow-up was performed, and the patient reported to be symptom-free. 

## 3. Discussion

The management of AF, particularly in patients with unique anatomical anomalies like the CIPV, presents consistent challenges. This case reinforces the significance of individualized treatment approaches in the management of AF, especially when dealing with rare anatomical variations. The successful outcome in this case was primarily attributable to utilizing advanced mapping technologies. These technologies played a crucial role in identifying and addressing the specific challenges of the patient’s unique PV anatomy. A comparative analysis of similar cases in the literature indicates that the precise application of such technologies is pivotal for achieving effective and safe ablation outcomes [8]. 

In patients with a CIPV anomaly, both inferior pulmonary veins drain via a common trunk. Consequently, there is a significantly increased likelihood for the esophagus to be in close proximity to the CIPV. The study conducted by Li et al. showed that in 44% of patients, the distance between the esophagus and CIPV was less than 5 mm [5]. Therefore, ablation with thermal energy exposes these patients to an increased risk of atrioesophageal fistula. However, in our case, the origin of AT during the second procedure was not in this area, so there was no additional risk, and esomeprazole (20 mg once daily) was prescribed for two months.

Intracardiac echocardiography (ICE) allows for easy visualization and navigation of devices and cardiac structures, such as the Vizigo sheath, ablation catheter, and PV ostium, during the procedure. This provides real-time data regarding contact, increasing efficacy and reducing the need for fluoroscopy [9]. ICE can be particularly important with regard to single-shot technology and in cases of similar PV anomalies. 

Recently, a study by Xie et al. highlighted the feasibility and safety of cryoballoon ablation in patients with CIPV [6]. Furthermore, the study by Li et al. reported that cryoballoon ablation is an efficient, safe, and time-saving technique with procedure outcomes similar to radiofrequency ablation [5]. A case report also demonstrates an effective ablation strategy combining 3D-electroanatomic mapping and pulsed-field ablation, which can be considered in challenging cases like CIPV. Considering pulsed field ablation allows for the selective ablation of atrial myocytes, this strategy can provide safer ablation [10]. However, in our experience, cryoballoon ablation in patients with anatomical variations in pulmonary veins can be challenging, and we prefer point-by-point ablation with 3D navigation.

To the best of our knowledge, there is no evidence that anomalies of pulmonary vein anatomy are associated with a higher incidence of atrial fibrillation or atrial tachycardia, neither in ablation-naive patients nor as a complication after previous PV isolation. In accordance with this, the reconnected PV was the right upper PV and not the anomalous common ostium of both lower veins, which presented as electrically silent, confirming that such abnormality is not essential for the origin of AT.

In the atrial remodeling theory of AF pathophysiology, any reentry requires a suitable vulnerable substrate and a trigger that acts on the substrate to initiate reentry. Ectopic firing contributes to reentry by providing triggers for reentry induction [11]. This substrate in the vein and a gap in the previous ablation line allow for regular transmission to the left atrium, resulting in AT. However, we do not have enough data to determine the exact mechanism of the tachycardia, as pacing maneuvers were not possible due to the very short tachycardia cycle length. The observed eccentric activation of the coronary sinus may be attributed to several factors. Notably, this could be due to suboptimal positioning with the decapolar catheter, particularly if the distal electrode pair was positioned in the posterolateral section of the coronary sinus, or it could be a result of localized areas of fibrosis.

The most common arrhythmia observed after radiofrequency ablation of pulmonary veins is atrial tachycardia, occurring in approximately 5–30% of cases [12]. Preventing unnecessary ablation lesions in the index procedure could help avoid creating a substrate for arrhythmogenesis. Due to prior AF ablation, atrial remodeling, and existing atrial myopathy altering the normal conduction of the atria, limitations exist when using ECG features, intracardiac tracings, or entrainment pacing to identify the circuit and accurately localize the critical isthmus. With the development of ultra-high-density mapping and novel annotation systems in electroanatomic mapping systems, clinical electrophysiologists can derive detailed information regarding the abnormal substrate and critical circuits of ATs, thereby effectively performing ablations without causing iatrogenic arrhythmias [12].

This case further highlights the need for personalized strategies in procedural approaches and techniques, considering each patient’s distinct anatomical characteristics. It also brings to light the potential for suboptimal outcomes in catheter ablation of AF in patients with paroxysmal AF when PV anatomy is variable. However, it must be noted that such anomalies do not necessarily have to be linked with the root cause of the origin of the arrhythmia. 

## 4. Conclusions

The presented case of repeated catheter ablation for AT in a patient with a CIPV anomaly showcases the importance of tailored interventions in complex anatomical scenarios. Long-term follow-up, marked by the absence of symptoms and documented sinus rhythm, emphasizes the sustained success of the procedure and its positive impact on the patient’s overall quality of life. Variable PV anatomy may, in part, explain the suboptimal outcome of catheter ablation of AF in some patients with paroxysmal AF. Despite the increased risk of complications, successful and safe RF ablation in these patients is achievable. This case contributes to the growing body of evidence supporting the feasibility and efficacy of catheter ablation in challenging anatomical conditions, providing valuable insights for future interventions.

## Data Availability

Data are contained within the article.

## References

[1] Calkins H., Hindricks G., Cappato R., Kim Y.-H., Saad E.B., Aguinaga L., Akar J.G., Badhwar V., Brugada J., Camm J. (2017). 2017 HRS/EHRA/ECAS/APHRS/SOLAECE expert consensus statement on catheter and surgical ablation of atrial fibrillation. Heart Rhythm.

[2] Hindricks G., Potpara T., Dagres N., Arbelo E., Bax J.J., Blomström-Lundqvist C., Boriani G., Castella M., Dan G.-A., Dilaveris P.E. (2021). 2020 ESC Guidelines for the diagnosis and management of atrial fibrillation developed in collaboration with the European Association for Cardio-Thoracic Surgery (EACTS). Eur. Heart J..

[3] Benali K., Lai V.D., Hammache N., Magnin-Poull I., de Chillou C., Sellal J.M. (2023). Impact of pulmonary veins anatomy on the outcomes of radiofrequency ablation for paroxysmal atrial fibrillation in the era of contact force-sensing ablation catheters. J Interv. Card. Electrophysiol..

[4] McLellan A.J.A., Ling L., Ruggiero D., Wong M.C.G., Walters T.E., Nisbet A., Shetty A.K., Azzopardi S., Taylor A.J., Morton J.B. (2014). Pulmonary vein isolation: The impact of pulmonary venous anatomy on long-term outcome of catheter ablation for paroxysmal atrial fibrillation. Heart Rhythm.

[5] Li J.H., Xie H.Y., Sun Q., Guo X.G., Chen Y.Q., Cao Z.J., Ma J. (2022). Comparison of Using Second-Generation Cryoballoon and Radiofrequency Catheter for Atrial Fibrillation Ablation in Patients With the Common Ostium of Inferior Pulmonary Veins. Front. Cardiovasc. Med..

[6] Xie H.Y., Guo X.G., Yang J.D., Li J.H., Chen Y.Q., Cao Z.J., Sun Q., Li X.Y., Ma J. (2021). Safety and Efficacy Using the Second-Generation Cryoballoon in Patients With Atrial Fibrillation and a Common Ostium of Inferior Pulmonary Veins. Front. Cardiovasc. Med..

[7] Kanaji Y., Miyazaki S., Iwasawa J., Ichihara N., Takagi T., Kuroi A., Nakamura H., Taniguchi H., Hachiya H., Iesaka Y. (2016). Pre-procedural evaluation of the left atrial anatomy in patients referred for catheter ablation of atrial fibrillation. J Cardiol..

[8] Canpolat U., Yorgun H., Hazirolan T., Aytemir K. (2021). Common ostium of inferior pulmonary veins: An extremely rare variant described by preprocedural computerized tomography angiography. J. Arrhythm..

[9] Maalouf J., Whiteside H.L., Pillai A., Omar A., Berman A., Saba S., Hreibe H. (2020). Reduction of radiation and contrast agent exposure in a cryoballoon ablation procedure with integration of electromagnetic mapping and intracardiac echocardiography: A single center experience. J. Interv. Card. Electrophysiol..

[10] Mittal A., Fitzpatrick N., Szeplaki G. (2023). Pulsed field ablation in common inferior pulmonary trunk. J. Interv. Card. Electrophysiol..

[11] Nattel S., Burstein B., Dobrev D. (2008). Atrial remodeling and atrial fibrillation: Mechanisms and implications. Circ. Arrhythm. Electrophysiol..

[12] Hung Y., Chang S.L., Lin W.S., Lin W.Y., Chen S.A. (2020). Atrial Tachycardias after Atrial Fibrillation Ablation: How to Manage?. Arrhythm. Electrophysiol. Rev..

